# Sex Differences in 24 H Blood Pressure in Night-Shift Workers

**DOI:** 10.3390/jcm14165728

**Published:** 2025-08-13

**Authors:** Barbara Toffoli, Silvia Berti, Ilaria Pitteri, Matilde Contessa, Federica Tonon, Rebecca Defendi, Andrea Grillo, Bruno Fabris, Stella Bernardi

**Affiliations:** 1 Department of Medical Surgical and Health Sciences, University of Trieste, Cattinara Teaching Hospital, Strada di Fiume 447, 34100 Trieste, Italy; btoffoli@units.it (B.T.); silvia.berti@studenti.units.it (S.B.); ilaria.pitteri@studenti.units.it (I.P.); matilde.contessa@asugi.sanita.fvg.it (M.C.); federica.tonon@asugi.sanita.fvg.it (F.T.); rebecca.defendi@studenti.units.it (R.D.); andrea.grillo@units.it (A.G.); b.fabris@fmc.units.it (B.F.); 2 Unit of Endocrinology (Medicina Clinica), ASUGI (Azienda Sanitaria Universitaria Giuliano Isontina), Cattinara Teaching Hospital, Strada di Fiume 447, 34100 Trieste, Italy

**Keywords:** blood pressure, hypertension, gender medicine, sex differences, night shift, shift work, circadian rhythm, clock genes, *BMAL1*

## Abstract

**Background/Objectives**: Gender Medicine addresses how sex- and gender-based differences influence people’s health. Blood pressure (BP), which is the leading global risk factor for cardiovascular disease, shows a sexual dimorphism. This is seen also in case of shift work, as shift work is associated with hypertension in the male sex. It is not clear if this may be extended also to night-shift work, as data for this are limited. Based on this background, the aim of this study was to evaluate whether there were sex differences in the 24 h BP profile during a day with a day shift and a day with a night shift. **Methods**: This study is a post hoc analysis of a previous study where we evaluated ambulatory blood pressure monitoring data for two days, in a day with a day shift (and night of rest) and in a day with a night shift. **Results**: Overall, 25 subjects (physicians) were included in the analysis, of whom 10 were (40%) males and 15 were (60%) females. No differences were recorded in terms of age, years of work, number of steps, admissions, and calls during the recordings between the two sexes. Subjects worked on average 1.2 night per month, indicating that this population had a low exposure to night-shift work. BP patterns did not differ between sexes, but BP levels were always higher in males than females. Nevertheless, only females showed a significant increase of SBP during the night shift as compared to the night of rest. Both males and females showed a significant reduction in BP dipping during the night shift, but only in females, the significant reduction in BP dipping was maintained after the night-shift work. Interestingly, *BMAL1* gene expression, which is a gene of the circadian rhythm, increased significantly after the night shift only in females, suggesting that females display an earlier acrophase than males after night-shift work, which may be associated with tolerance to shiftwork. **Conclusions**: Our data are consistent with the concept that blood pressure display several sex/gender differences. Males had higher BP values, but females showed signs of lower tolerance to shift work. This might be due to sex differences in the circadian rhythm regulation, which, in turns, regulate physiological functions, such as blood pressure.

## 1. Introduction

The World Health Organization defines Gender Medicine, or rather Gender-specific Medicine, as the study of how (sex-based) biological, (gender-based) socioeconomic, and cultural differences influence people’s health. Biological sex stems from chromosome asset, i.e., XY in males and XX in females, and it is determined by genetic and hormonal influences, which, in turn, modify disease pathophysiology, clinical manifestations, and response to treatment [1]. On the other hand, gender, according to the Global Health 50/50 definition, refers to the socially constructed norms that impose and determine roles, relationships, and positional power for all people across their lifetime [2]. Sex and gender are distinct concepts, but their influence on health outcomes is often linked [3]. Scientific evidence shows that gender equality in science, medicine, and global health matters for health and health-related outcomes, and it has the potential to lead to substantial health, social, and economic gains [2].

Hypertension is the leading global risk factor for cardiovascular disease (CVD) and mortality [3]. Since 1990, the number of people with hypertension has doubled, with most of the increase occurring in low-income and middle-income regions. Prevalence of hypertension does not show a sex/gender difference, as it is esteemed to be 32% for women and 34% for men [3]. Nevertheless, blood pressure (BP) trajectories over the life course differ between sexes/genders, as in adulthood, men have higher BP values than women; then, after menopause, BP values rise sharply in women but not in men [4,5]. Second, CVD risk begins at lower thresholds of systolic BP for women than for men [6]. For instance, a study that included 1.25 million patients and 11029 myocardial infarction events found a slightly higher relative risk (RR) in women than in men of myocardial infarction with increasing systolic blood pressure. Third, the response to treatment [7,8,9] as well as the type and prevalence of additional CVD risk factors show a gender difference [4]. The particularities of arterial hypertension in the female sex have been recently reviewed by Lazaridis et al. [10].

It has been recently shown that also the BP trajectories during a 24 hour (24 h) cycle differ between the two sexes. Omboni et al. have shown that a blunted nocturnal BP fall and an abnormal morning BP rise were more common in males, while higher daytime BP and HR variability were more common in females, as assessed by ambulatory blood pressure monitoring [11]. We have recently reported that shift work significantly changes blood pressure profiles, as it increases night systolic blood pressure, night diastolic blood pressure, and heart rate, with a parallel decrease of their physiologic nocturnal decline [12]. Although shift work has been associated with hypertension in men [13], it is unknown if this applies also to night-shift work [14]. Based on this background, in this post hoc analysis, we examined whether there were sex differences in the 24 h BP profile during a day with a day shift and a day with a night shift.

## 2. Materials and Methods

### 2.1. Study Design

This study is a post hoc analysis of a previous work [12], where we evaluated ambulatory blood pressure monitoring(ABPM) data for two days, a day with a day shift and a day with a night shift. The aim of this study was to analyze the impact of shift work on BP in males and females, evaluating whether there were sex differences in the 24 h BP profile during a day with a day shift and a day with a night shift.

Details of the study design are published elsewhere [12]. Briefly, in this study we enrolled physicians eligible for night-shift work in the Internal Medicine Department of our hospital (Cattinara Teaching Hospital, Azienda Sanitaria Universitaria Giuliano Isontina ASUGI, Trieste, Italy). Inclusion criteria were age between 25 and 60 years; night-shift work eligibility; and consent to take part in the study. Exclusion criteria were history of any acute or chronic disease; children aged < 2 years; and holidays in the 3 weeks prior to study measurements.

After providing informed consent, each subject (physician) wore an ABPM device twice. First, they wore it during a control day (cnt) that included a day shift (from 08 to 20) and 12 h of rest (from 20 to 08). Second, they wore it on a day with a night shift (ns) that included 12 h of rest (from 08 to 20), 12 h of night shift (from 20 to 08), and 6 h of rest after the night shift (from 08 to 13). ABPM was performed with a clinically validated automated electronic upper arm oscillometric device (SpaceLabs Oscillometric Model 90217 (SpaceLabs Healthcare, Snoqualmie, WA, USA). The device was programmed to take a reading every 20 min.

Before ABPM, we recorded the medical history and the anthropometric parameters of each participant. During monitoring, participants were asked to complete a diary where daily activities and sleep time had to be reported. Study participants were also asked to fast from 24 to 8. After ABPM termination, participants underwent blood sampling to measure cytokines and to evaluate clock gene expression.

The study was conducted in accordance with the Helsinki Declaration, and the protocol was approved by the Institutional Review Board and Ethics Committee (Comitato Etico Unico Regionale CEUR 107_2020H) on 6 December 2022.

### 2.2. Peripheral Blood Mononuclear Cell Isolation and Clock Gene Expression Analyses

Isolation of peripheral blood mononuclear cells (PBMCs), RNA extraction, cDNA synthesis, and gene expression analysis via RT-qPCR were carried out as previously described [12,15]. Primer sequences used with the SYBR Green System are reported in Table 1.

### 2.3. Statistical Analyses

All statistical analyses were carried out in the R system for statistical computing (Version 4.0.2; R development Core Team, 2020). Statistical significance was set at *p* < 0.05. The Shapiro–Wilk test was applied to continuous variables to check for distribution normality. Quantitative variables were reported as the median with the IQR. Categorical variables were reported as absolute frequencies and/or percentages. Continuous variables were compared by the Mann–Whitney test (and Kruskall–Wallis test) or Student’s *t*-test (and ANOVA), depending on data distribution and the number of groups.

## 3. Results

### 3.1. Characteristics of the Study Population

Overall, 25 subjects were included in the analysis, of whom 10 (40%) were males and 15 (60%) were females. They were all physicians eligible to night-shift work, working in the same Department of Internal Medicine. Given that these subjects had the same cultural background, the same level of education, and type of work, we decided to use the terms sex, males, and females rather than the terms gender, men, and women.

All subjects had a Caucasian background. No differences were recorded in terms of age (males had a median age of 33 years, and females had a median age of 31 years), years of work, number of night shifts/month, number of steps, admissions, and calls during the recordings. Males had higher BMI, which was 24.6, as compared to females, whose BMI was 22. Only four female physicians had history of previous pregnancies, but none of them had history of gestational hypertension, which has been linked to poor sleep quality [16]. The characteristics of the study population are reported in Table 2.

### 3.2. Blood Pressure Patterns and Levels in the Whole Study Population and According to Sex

**Figure 1:**
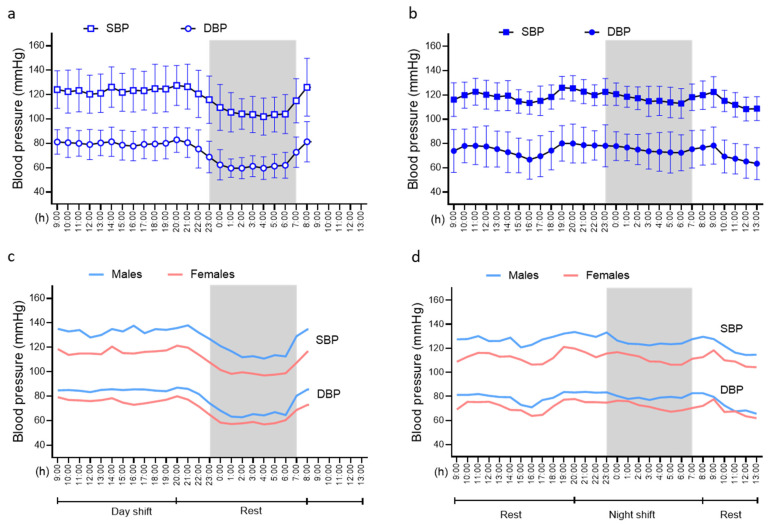
Blood pressure values (hourly averages) in the entire cohort as well as in males and females. (**a**) Hourly averages of 24 h ABPM during a day with a day shift (8–20) and a night of rest (20–8), cnt day. (**b**) Hourly averages of 24 h ABPM during a day with a day of rest (8–20) and a night shift (20–8) with the subsequent period of rest (08–13), ns day. (**c**) Hourly averages of 24 h ABPM in the cnt day in males and females. (**d**) Hourly averages of 24 h ABPM in the ns day in males and females. Gray areas correspond to the usual sleep time.

Figure 1a,b show hourly averages of systolic blood pressure (SBP) and diastolic blood pressure (DBP) during two consecutive ABPM recordings. The first round of ABPM (Figure 1a) was performed during a day with a day shift at work and a night of rest. Here, SBP and DBP showed a typical circadian pattern, with higher values during the waking hours and lower values during the night sleep. The second round of ABPM (Figure 1b) was performed during a day with a day of rest and a night shift at work and a subsequent period of rest (09–13). Here, SBP and DBP did not show the typical nocturnal decline, but lower values were seen during the rest hours after the night shift.

Figure 1c,d show hourly averages of SBP and DBP during the ABPM recordings in males and females. BP patterns did not differ between sexes, but BP levels were always higher in males than females in both recordings.

**Figure 2:**
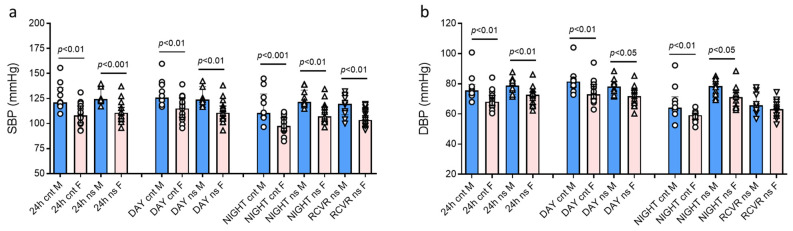
The 24 h, daytime, and nighttime blood pressure values in males and females. (**a**) Systolic blood pressure values; (**b**) diastolic blood pressure values. Blood pressure values are presented as median (top of the histogram) with interquartile ranges. “F” is for female group, “M” is for male group, “cnt” is for control day (day shift at work and night of rest), “ns” is for night-shift day (day of rest, night shift at work, subsequent rest), “24h” is for 24 h, “DAY” is for the daytime (9–23), “NIGHT” is for the nighttime (sleep) (23–7), and “RCVR” is for the time of rest after the night shift (9–13).

Figure 2a,b show SBP and DBP values during 24 h as well as in the daytime and nighttime periods. Both SBP and DBP values were always higher in males than females during the 24 h, daytime, and nighttime periods, except for the rest after the night shift.

### 3.3. Paired Analysis of Blood Pressure at Night (Rest vs. Work) in Males and Females

**Figure 3:**
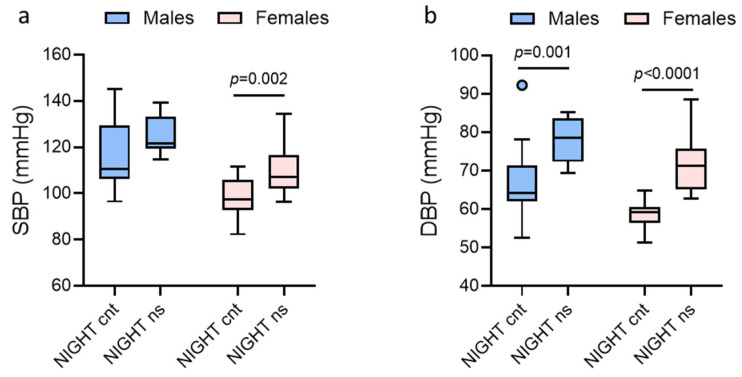
Paired analysis of blood pressure at night (rest vs. work) in males and females. (**a**) Systolic blood pressure (SBP). (**b**) Diastolic blood pressure (DBP) dippings. Data are presented as standard box plots. “NIGHT” is for nighttime, “cnt” is for control day (day shift at work and night of rest), and “ns” is for night-shift day (day of rest and night shift at work).

Figure 3a,b show the paired analysis of SBP and DBP at night (rest vs. work) in males and females. SBP increased significantly during the night shift in females but not in males, while DBP increased significantly during the night shift in both sexes.

### 3.4. Blood Pressure Dippings in Males and Females

**Figure 4:**
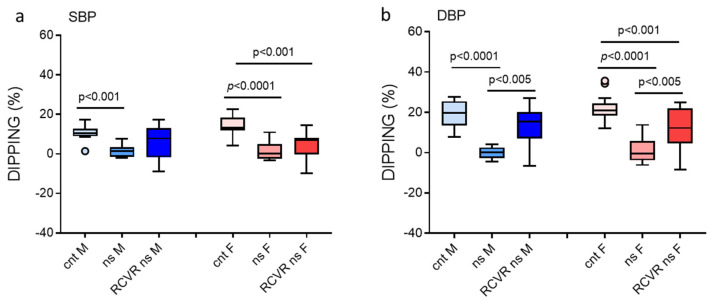
Blood pressure dippings in males and females. Dippings are the % reduction in blood pressure at night/rest with respect to the day/wake period. (**a**) Systolic blood pressure (SBP) dippings. (**b**) Diastolic blood pressure (DBP) dippings. Data are presented as standard box plots. “F” is for female group, “M” is for male group, “cnt” is for control day (day shift at work and night of rest), “ns” is for night-shift day (day of rest and night shift at work), and “RCVR” is for the rest after the night shift.

Blood pressure has a circadian rhythm, and at night, it generally decreases by 10–20%, which is known as dipping. Loss of this blood pressure decrease, known as non-dipping, is associated with metabolic and cardiovascular diseases [17,18]. Figure 4a,b show SBP and DBP dippings, during the night of the cnt day, the night of the ns day, and the rest after the night shift. In particular, both males and females showed a significant reduction in BP dippings during the night shift. The significant reduction in BP dipping was maintained in the period of rest after the night shift in females only.

The magnitude of the nocturnal SBP dipping in males was 10.57 (9.10–11.19) % during a night of rest (cnt), while it decreased to 1.46 (−1.39–2.96) % during a night of work (ns) and to 7.83 (0.06–12.10) % during the rest after night-shift work (RCVR). The magnitude of the nocturnal SBP dipping in females was 13.27 (12.03–16.16) % during a night of rest (cnt), while it decreased to 0.27 (−1.62–4.77) % during a night of work (ns) and to 7.02 (2.16–8.05) % during the rest after night-shift work (RCVR). The magnitude of the nocturnal DBP dipping in males was 19.61 (14.20–23.80) % during a night of rest (cnt), while it decreased to 0.18 (−2.11–2.15) % during a night of work (ns) and to 15.47 (11.74–20.01) % during the rest after night-shift work (RCVR). The magnitude of the nocturnal DBP dipping in females was 21.05 (18.73–23.45) % during a night of rest (cnt), while it decreased to −0.41 (−3.10–3.59) % during a night of work (ns) and to 12.28 (5.03–20.93) % during the rest after night-shift work (RCVR).

### 3.5. Circadian Rhythm Gene Expression in Males and Females

Our normal 24 h circadian rhythm is based on a master clock that resides in the suprachiasmatic nucleus of the hypothalamus, which is entrained by light, and on peripheral clocks that integrate signals coming from the master clock as well as the periphery, such as light and food. These clocks regulating circadian rhythm rely on cellular networks of transcription factors (core clock genes) that control circadian variations in cellular gene expression, which, in turn, regulate most physiological functions over 24 h, such as blood pressure [19]. Clocks are based on four core clock proteins—Brain and Muscle ARNT-like 1 (*BMAL1*), Circadian Locomotor Output Cycles Protein Kaput (CLOCK), Cryptochrome (CRY), and Period (PER)—that act as transcription factors to regulate nearly 50% of genes in mammals [20]. *BMAL1* and CLOCK are part of the positive arm of the clock, peaking during the day. They form a heterodimer and bind to E-box response elements to induce transcription of target genes, which include *CRY* and *PER*. CRY and PER, which peak at night, act in the negative arm to inhibit the action of *BMAL1*/*CLOCK* [20].

**Figure 5:**
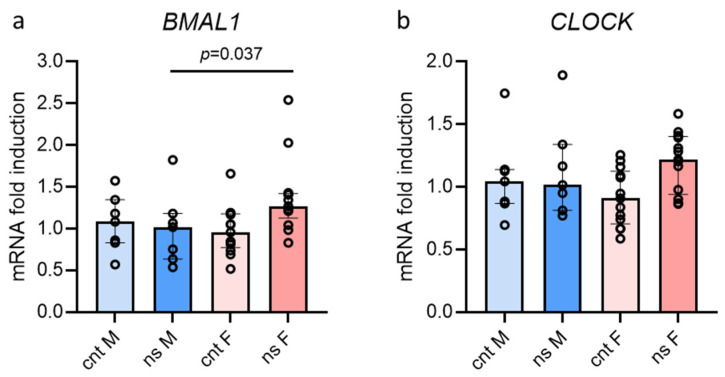
Circadian rhythm gene expression in males and females. Gene expression after a night of rest (cnt) vs. after a night shift (ns). (**a**) *BMAL1*; (**b**) *CLOCK*. Data are presented as median with interquartile ranges. “F” is for female group; “M” is for male group.

Figure 5a,b show *BMAL1* and *CLOCK* gene expression in PBMCs after a night of rest (cnt) and a night shift (ns) in males and females. Interestingly, our data show that *BMAL1* gene expression increased significantly after the night shift in females but not in males, indicating that the night shift had a greater impact on the circadian rhythm in females, who display an earlier acrophase as compared to males. Also, CLOCK gene expression increased although not significantly, while we did not find any difference in the expression of the other genes explored.

## 4. Discussion

First of all, our data show that 24 h BP patterns did not differ between sexes, but BP levels were always higher in males than females. These data are consistent with the study by Omboni et al., who analyzed the ABPM recordings of 52,911 subjects, finding that females had a more favorable 24 h BP profile than males [11]. In addition, our data extends the findings by Omboni et al. to young and healthy subjects with no hypertension or significant comorbidities. In particular, in the study by Omboni et al., participants were aged, on average, 57 years and 35% of them suffered from hypertension, 11% from CVD, and 3% from diabetes [11]. By contrast, in our study, ABPM was performed on healthy subjects (physicians eligible for night-shift work), aged, on average, 33 years, with no history of hypertension or other diseases. In general, our data are also in line with the National Health and Nutrition Examination Survey (NHANES) reports, demonstrating that from early adulthood to the age of 60 years the mean values of BP are higher in men than women and that hypertension is more common among men than women [21,22]. By contrast, the same NHANES reports show that after the sixth decade of life, the incidence of hypertension increases more rapidly in women than in men, with the prevalence of hypertension in women exceeding that of men.

This sexual dimorphism in BP levels and changes over time seems to be due not only to sex hormones but also to sex chromosomes, as reviewed by Colafella et al. [23]. On one hand, endogenous estrogens are associated with lower BP in women. Animal models have shown that ovariectomy raised baseline BP, and this was reversed by 17βestradiol [24]. Not surprisingly, from a clinical point of view, the risk of cardiovascular disease is higher in women who had premature menopause (age < 40 years), early menopause (age 40–44 years), and relatively early menopause (age 45–49 years) as compared with women who had menopause at age 50–51 years [25]. This BP-lowering effect of estrogens include their impact on the renin–angiotensin–aldosterone system (RAAS) and the endothelin system. With respect to the RAAS, scientific evidence indicate that estrogens shift the balance towards the protective vasodilating arm of the RAAS (ACE2, Angiotensin 1-7, AT2, and Mas receptors) [26]. In line with this concept, we demonstrated that ovariectomy was associated with an upregulation of the genes of the vasoconstrictor arm of the RAAS (ACE and (AT1) receptors) [27]. On the other hand, the impact of sex on BP depends not only on hormones but also on the differential expression of sex chromosomal genes. This is due to the fact that in males, there are genes unique to the Y chromosome, and in females, 12–20% of genes may escape the X chromosome inactivation, which makes certain genes more highly expressed in females than in males. Interestingly, genes encoding for components of the protective arm of the RAAS, such as the AT2 receptor or ACE2, are located on the X chromosome, and we have recently demonstrated that they are more expressed in PBMCs of healthy women than men [28].

The second finding of our study is that although BP levels were always higher in males than females, only females had a significant increase in SBP during the night shift as compared to the night of rest. In addition, both males and females showed a significant reduction in SBP and DBP dippings during the night shift, but this reduction was maintained after the night-shift work only in females. This is in line with the notion that there are sex differences in tolerance to shift work [29]. For instance, previous studies have shown that female shift workers have more sleep-related issues than males [30], and in line with our data, Rotenberg et al. found that female shift workers had a shorter sleep duration on the first episode of rest/sleep after the night shift as compared to males [31].

The question that remains unanswered is whether the shorter sleep duration and reduced BP dipping after the night shift in females is a sex or gender issue. One might argue that this difference is a gender-driven feature, as women coming back from work are more likely to start household activities. On the other hand, though, our data suggest that this might be a sex-driven feature instead, given that *BMAL1* gene expression in peripheral blood mononuclear cells increased significantly at the end of the night shift in females but not in males. This is consistent with the findings of Costello et al. showing that *BMAL1* and other clock genes were more highly expressed in the kidneys of female mice at 6 am [32]. The authors concluded that young females seem to have more robust peripheral clocks, and this might contribute to sex differences in circadian clock function [32]. Other works have shown that men are more evening oriented than women [33] and that acrophase timing displays variation with sex [34].

Nevertheless, the differences in sleep between the two sexes may not translate into a difference in the risk of cardiovascular morbidity or mortality. In other words, even if female shift workers seem to have more frequent difficulties falling asleep, higher SBP increase during the night shift, shorter sleep duration, and reduced BP dipping after the night shift, the literature indicates that males have higher risk of health problems. In a systematic review and meta-analysis aiming to assess the association between shift work status and hypertension, Manohar et al. found a significant association between rotating shiftwork and hypertension in male workers, while there was no specific association between night-shift work and hypertension due to the lack of data [13]. Consistent with this, a recent study on a population with low exposure to night-shift work (female and male workers worked on average 1.7 and 1.8 night shifts per month for less than 4 years) has provided evidence for a 20% increased risk of coronary heart disease among men but not among women [35].

It has to be noted that age is also an important factor influencing sex-based differences in coronary artery disease, because women are generally older (>60 years) when they present a first event [36], and this might affect the data on night-shift work and CVD risk between sexes/genders. Likewise, when evaluating the relationship between night-shift work and cardiovascular morbidity and mortality, not only the number of night shifts per months but also the length of night shift work duration must be taken into account. For instance, recently, Wang et al. showed that both cumulative night shift work duration (≥10 years) as well as the monthly frequency of night shifts (3–8 nights/month) were associated with increased atrial fibrillation risk as well as coronary heart disease [37]. Interestingly, in this study, there was a stronger association between lifetime duration of night-shift work and atrial fibrillation in women than men [37].

Strengths and limitations. The strengths of this study include the population characteristics, as we selected a relatively healthy, young, and homogenous cohort—Caucasian physicians with low exposure to night work—minimizing confounding from comorbidities and medications, as the subjects did not suffer from relevant comorbidities. Secondly, the use of ambulatory blood pressure monitoring (ABPM) simultaneously on two consecutive days, comparing a day shift versus a night shift with subsequent rest, allowed for paired within-subject analyses, enhancing the reliability of BP comparisons. Third, in this study, we combined the assessment of clinical parameters with gene expression analysis to get a better overview of the impact of night-shift work on blood pressure and circadian rhythm in the two sexes/genders. Having said that, the limitations of this study include the number of participants and their characteristics. In particular, this study had a small sample size (*n* = 25) with an uneven sex distribution (15 females and 10 males), which might limit the generalizability of findings. In addition, the study population characteristics (young age, same ethnic background, as well as same work, hospital, and night-shift exposure) may not reflect broader shift-working populations with older ages, different backgrounds, occupations, or higher night-shift loads. Last, clock gene expression was evaluated only in the mornings and only in PBMCs, and the study design did not include longitudinal follow-ups.

## 5. Conclusions

Our data are consistent with the concept that blood pressure display several sex/gender differences. Males had higher BP values, but females showed signs of lower tolerance to shift work, such as greater SBP increases during the night shift and reduced BP dipping during the period of rest after the night shift. This was associated with an upregulation of *BMAL1* after the night shift. This suggests that sex differences in circadian rhythm regulation, which regulates physiological functions, might impact blood pressure changes during night shifts as well as tolerance to shift work. Further studies are needed to clarify if sex differences in BP during and after the night shifts are associated with a difference in the risk of cardiovascular morbidity or mortality

## Figures and Tables

**Table 1:** Primer sequences.

Target (GenBank Accession Number)	Primer Pair	Mature Transcript	Amplicon Size (bp)
*BMAL1* (NM_001297719)	(F) 5′-TTACTGTGCTAAGGATGGCTG-3′ (R) 5′-GCCCTGAGAATGAGGTGTTTC-3′	(F) 731–751 (R) 857–837	127
*CLOCK* (NM_004898)	(F) 5′-CTACATTCACTCAGGACAGGC-3′ (R) 5′-GGCCCATAAGCATAGTACTAGG-3′	(F) 2495–2515 (R) 2614–2593	120
*PER1* (NM_002616)	(F) 5′-CCTCCAGTGATAGCAACGG-3′ (R) 5′-ACCAGATGCACATCCTTACAG-3′	(F) 1784–1802 (R) 1874–1854	91
*PER2* (NM_022817)	(F) 5′-GCCAGAGTCCAGATACCTTTAG-3′ (R) 5′-TGTGTCCACTTTCGAAGACTG-3′	(F) 505–526 (R) 602–582	98
*PER3* (NM_001377275)	(F) 5′-CTGTCTCACTGTGGTTGAAAAG-3′ (R) 5′-TAGGTAACCCAGCAAAGGC-3′	(F) 1063–1084 (R) 1207–1189	145
*CRY1* (NM_004075)	(F) 5′-TCCCGTCTGTTTGTGATTCG-3′ (R) 5′-TTAATAGCTGCGTCTCGTTCC-3′	(F) 799–818 (R) 929–909	131
*CRY2* (NM_021117)	(F) 5′-TGGATAAGCACTTGGAACGG-3′ (R) 5′-AGAGACAACCAAAGCGCAG-3′	(F) 735–754 (R) 854–836	120

**Table 2:** Characteristics of the study population according to sex.

Variable		Entire Cohort (*n* = 25)	Males (*n* = 10)	Females (*n* = 15)	*p* Value
Age		33 (29–37)	33 (31–36)	31 (29–37)	0.867
BMI		22.6 (20.8–24.5)	24.6 (22.0–26.9)	22.1 (20.3–23.0)	0.037
Physical activity	Yes (%) No (%)	12 (48%) 13 (52%)	3 (30%) 7 (70%)	9 (60%) 6 (40%)	0.226
Medical conditions	Yes (%) No (%)	3 (12%) 22 (88%)	2 (20%) 8 (80%)	1 (7%) 14 (93%)	0.543
Medication	Yes (%) No (%)	5 (20%) 20 (80%)	2 (20%) 8 (80%)	3 (20%) 12 (80%)	1
Years of work		7 (2–10)	7 (5–9)	4 (2–11)	0.636
Working position	Attending (%) Resident (%)	13 (52%) 12 (48%)	4 (40%) 6 (60%)	9 (60%) 6 (40%)	0.428
Day shift steps		7720 (6504–11,062)	6940 (5881–12,191)	8000 (6944–10,307)	0.591
Night shift/month		1.2 (1–2)	1.2 (1–2)	1.2 (1–2)	1
Night-shift calls		17.5 (13–20)	18 (11.5–20)	17.5 (15–19)	0.893
Night-shift admissions		8.5 (6–10)	8 (6–9)	9 (6.5–10)	0.567
Night-shift steps		6660 (5785–7822)	7414 (6521–8291)	6436 (5603–7369)	0.209

Data are expressed as median (IQR). BMI is for body mass index.

## Data Availability

The original contributions presented in this study are included in the article. Further inquiries can be directed to the corresponding author(s).

## References

[1] Mauvais-Jarvis F., Bairey Merz N., Barnes P.J., Brinton R.D., Carrero J.J., DeMeo D.L., De Vries G.J., Epperson C.N., Govindan R., Klein S.L. (2020). Sex and gender: Modifiers of health, disease, and medicine. Lancet.

[2] Shannon G., Jansen M., Williams K., Caceres C., Motta A., Odhiambo A., Eleveld A., Mannell J. (2019). Gender equality in science, medicine, and global health: Where are we at and why does it matter?. Lancet.

[3] Collaboration N.C.D.R.F. (2021). Worldwide trends in hypertension prevalence and progress in treatment and control from 1990 to 2019: A pooled analysis of 1201 population-representative studies with 104 million participants. Lancet.

[4] Gerdts E., Sudano I., Brouwers S., Borghi C., Bruno R.M., Ceconi C., Cornelissen V., Dievart F., Ferrini M., Kahan T. (2022). Sex differences in arterial hypertension. Eur. Heart J..

[5] Ji H., Kim A., Ebinger J.E., Niiranen T.J., Claggett B.L., Bairey Merz C.N., Cheng S. (2020). Sex Differences in Blood Pressure Trajectories Over the Life Course. JAMA Cardiol..

[6] Ji H., Niiranen T.J., Rader F., Henglin M., Kim A., Ebinger J.E., Claggett B., Merz C.N.B., Cheng S. (2021). Sex Differences in Blood Pressure Associations With Cardiovascular Outcomes. Circulation.

[7] Bidel Z., Nazarzadeh M., Canoy D., Copland E., Gerdts E., Woodward M., Gupta A.K., Reid C.M., Cushman W.C., Wachtell K. (2023). Sex-Specific Effects of Blood Pressure Lowering Pharmacotherapy for the Prevention of Cardiovascular Disease: An Individual Participant-Level Data Meta-Analysis. Hypertension.

[8] Olivieri O., Pizzolo F., Ciacciarelli A., Corrocher R., Signorelli D., Falcone S., Blengio G.S. (2008). Menopause not aldosterone-to-renin ratio predicts blood pressure response to a mineralocorticoid receptor antagonist in primary care hypertensive patients. Am. J. Hypertens..

[9] Bager J.E., Manhem K., Andersson T., Hjerpe P., Bengtsson-Bostrom K., Ljungman C., Mourtzinis G. (2023). Hypertension: Sex-related differences in drug treatment, prevalence and blood pressure control in primary care. J. Hum. Hypertens..

[10] Lazaridis A., Malliora A., Gkaliagkousi E. (2025). The Particularities of Arterial Hypertension in Female Sex: From Pathophysiology to Therapeutic Management. J. Clin. Med..

[11] Omboni S., Khan N.A., Kunadian V., Olszanecka A., Schutte A.E., Mihailidou A.S. (2023). Sex Differences in Ambulatory Blood Pressure Levels and Subtypes in a Large Italian Community Cohort. Hypertension.

[12] Toffoli B., Tonon F., Giudici F., Ferretti T., Ghirigato E., Contessa M., Francica M., Candido R., Puato M., Grillo A. (2023). Preliminary Study on the Effect of a Night Shift on Blood Pressure and Clock Gene Expression. Int. J. Mol. Sci..

[13] Manohar S., Thongprayoon C., Cheungpasitporn W., Mao M.A., Herrmann S.M. (2017). Associations of rotational shift work and night shift status with hypertension: A systematic review and meta-analysis. J. Hypertens..

[14] Costello H.M., Gumz M.L. (2021). Circadian Rhythm, Clock Genes, and Hypertension: Recent Advances in Hypertension. Hypertension.

[15] Tonon F., Tornese G., Giudici F., Nicolardi F., Toffoli B., Barbi E., Fabris B., Bernardi S. (2022). Children With Short Stature Display Reduced ACE2 Expression in Peripheral Blood Mononuclear Cells. Front. Endocrinol..

[16] Wang T.L., Bryan S.G., Jeyabalan A., Facco F.L., Gandley R.E., Hubel C.A., Catov J.M., Hauspurg A.K. (2024). Sleep Quality in Individuals with and without Persistent Postpartum Hypertension. Am. J. Perinatol..

[17] Huart J., Persu A., Lengele J.P., Krzesinski J.M., Jouret F., Stergiou G.S. (2023). Pathophysiology of the Nondipping Blood Pressure Pattern. Hypertension.

[18] Ohkubo T., Imai Y., Tsuji I., Nagai K., Watanabe N., Minami N., Kato J., Kikuchi N., Nishiyama A., Aihara A. (1997). Relation between nocturnal decline in blood pressure and mortality. The Ohasama Study. Am. J. Hypertens..

[19] Huang R.C. (2018). The discoveries of molecular mechanisms for the circadian rhythm: The 2017 Nobel Prize in Physiology or Medicine. Biomed. J..

[20] Cox K.H., Takahashi J.S. (2019). Circadian clock genes and the transcriptional architecture of the clock mechanism. J. Mol. Endocrinol..

[21] Burt V.L., Whelton P., Roccella E.J., Brown C., Cutler J.A., Higgins M., Horan M.J., Labarthe D. (1995). Prevalence of hypertension in the US adult population. Results from the Third National Health and Nutrition Examination Survey, 1988-1991. Hypertension.

[22] Benjamin E.J., Blaha M.J., Chiuve S.E., Cushman M., Das S.R., Deo R., de Ferranti S.D., Floyd J., Fornage M., Gillespie C. (2017). Heart Disease and Stroke Statistics-2017 Update: A Report From the American Heart Association. Circulation.

[23] Colafella K.M.M., Denton K.M. (2018). Sex-specific differences in hypertension and associated cardiovascular disease. Nat. Rev. Nephrol..

[24] Hernandez I., Delgado J.L., Diaz J., Quesada T., Teruel M.J., Llanos M.C., Carbonell L.F. (2000). 17beta-estradiol prevents oxidative stress and decreases blood pressure in ovariectomized rats. Am. J. Physiol. Regul. Integr. Comp. Physiol..

[25] Zhu D., Chung H.F., Dobson A.J., Pandeya N., Giles G.G., Bruinsma F., Brunner E.J., Kuh D., Hardy R., Avis N.E. (2019). Age at natural menopause and risk of incident cardiovascular disease: A pooled analysis of individual patient data. Lancet Public Health.

[26] Tikellis C., Bernardi S., Burns W.C. (2011). Angiotensin-converting enzyme 2 is a key modulator of the renin-angiotensin system in cardiovascular and renal disease. Curr. Opin. Nephrol. Hypertens..

[27] Fabris B., Candido R., Bortoletto M., Toffoli B., Bernardi S., Stebel M., Bardelli M., Zentilin L., Giacca M., Carretta R. (2011). Stimulation of cardiac apoptosis in ovariectomized hypertensive rats: Potential role of the renin-angiotensin system. J. Hypertens..

[28] Tonon F., Candido R., Toffoli B., Tommasi E., Cortello T., Fabris B., Bernardi S. (2022). Type 1 diabetes is associated with significant changes of ACE and ACE2 expression in peripheral blood mononuclear cells. Nutr. Metab. Cardiovasc. Dis..

[29] Saksvik I.B., Bjorvatn B., Hetland H., Sandal G.M., Pallesen S. (2011). Individual differences in tolerance to shift work--a systematic review. Sleep. Med. Rev..

[30] Marquie J.C., Foret J. (1999). Sleep, age, and shiftwork experience. J. Sleep. Res..

[31] Rotenberg L., Portela L., Marcondes W.B., Moreno C., Nasciemento C.P. (2000). Gender and diurnal sleep in night workers at Brazilian industry. Shift Work 21st Century Arb. Der Betrieblichen Prax..

[32] Costello H.M., Eikenberry S.A., Cheng K.Y., Broderick B., Joshi A.S., Scott G.R., McKee A., Mendez V.M., Douma L.G., Crislip G.R. (2025). Sex differences in the adrenal circadian clock: A role for BMAL1 in the regulation of urinary aldosterone excretion and renal electrolyte balance in mice. Am. J. Physiol. Ren. Physiol..

[33] Randler C., Engelke J. (2019). Gender differences in chronotype diminish with age: A meta-analysis based on morningness/chronotype questionnaires. Chronobiol. Int..

[34] Shim J., Fleisch E., Barata F. (2024). Circadian rhythm analysis using wearable-based accelerometry as a digital biomarker of aging and healthspan. NPJ Digit. Med..

[35] Vestergaard J.M., Dalboge A., Bonde J.P.E., Garde A.H., Hansen J., Hansen A.M., Larsen A.D., Harma M., Costello S., Bottcher M. (2023). Night shift work characteristics and risk of incident coronary heart disease among health care workers: National cohort study. Int. J. Epidemiol..

[36] Canto J.G., Rogers W.J., Goldberg R.J., Peterson E.D., Wenger N.K., Vaccarino V., Kiefe C.I., Frederick P.D., Sopko G., Zheng Z.J. (2012). Association of age and sex with myocardial infarction symptom presentation and in-hospital mortality. JAMA.

[37] Wang N., Sun Y., Zhang H., Wang B., Chen C., Wang Y., Chen J., Tan X., Zhang J., Xia F. (2021). Long-term night shift work is associated with the risk of atrial fibrillation and coronary heart disease. Eur. Heart J..

